# Association Between Female Genital Schistosomiasis and High-Risk Human Papillomavirus Among Women of Reproductive Age in Zambia: The Schista Study

**DOI:** 10.1093/infdis/jiag153

**Published:** 2026-03-13

**Authors:** Olimpia Lamberti, Rhoda Ndubani, Jennifer Fitzpatrick, Emily Webb, Nkatya Kasese, Beatrice Nyondo, Barry Kosloff, Maina Cheeba, Philippe Mayaud, Morgan E Lemin, Pytsje T Hoekstra, Govert Van Dam, Paul Corstjens, Lisette Van Lieshout, Bonnie Webster, Helen Ayles, Isaiah Hansingo, Bodo Randrianasolo, Dingase Kuwemba, Bellington Vwalika, Paul Kamfwa, Kwame Shanaube, Helen Kelly, Amaya L Bustinduy

**Affiliations:** Department of Clinical Research, London School of Hygiene and Tropical Medicine, London, United Kingdom; Zambart, Lusaka, Zambia; Zambart, Lusaka, Zambia; Department of Infectious Disease Epidemiology, London School of Hygiene and Tropical Medicine, London, United Kingdom; Zambart, Lusaka, Zambia; Zambart, Lusaka, Zambia; Zambart, Lusaka, Zambia; Longhorn Vaccines and Diagnostics LLC, Bethesda, Maryland, USA; Zambart, Lusaka, Zambia; Department of Clinical Research, London School of Hygiene and Tropical Medicine, London, United Kingdom; Department of Clinical Research, London School of Hygiene and Tropical Medicine, London, United Kingdom; Leiden University Centre for Infectious Diseases, Leiden University Medical Centre, Leiden, The Netherlands; Leiden University Centre for Infectious Diseases, Leiden University Medical Centre, Leiden, The Netherlands; Department of Cell and Chemical Biology, Leiden University Medical Centre, Leiden, The Netherlands; Leiden University Centre for Infectious Diseases, Leiden University Medical Centre, Leiden, The Netherlands; Department of Science, Natural History Museum, London, United Kingdom; Department of Clinical Research, London School of Hygiene and Tropical Medicine, London, United Kingdom; Zambart, Lusaka, Zambia; Livingstone University Teaching Hospital, Livingstone, Zambia; Association K’OLO VANONA, Antananarivo, Madagascar; Centre for Health, Agriculture and Development Research and Consulting (CHAD), Blantyre, Malawi; University of Zambia, Lusaka, Zambia; University Teaching Hospital, Cancer Disease Hospital, Lusaka, Zambia; Zambart, Lusaka, Zambia; Department of Clinical Research, London School of Hygiene and Tropical Medicine, London, United Kingdom; School of Public Health, Physiotherapy and Sports Science, University College Dublin, Ireland; Department of Clinical Research, London School of Hygiene and Tropical Medicine, London, United Kingdom

**Keywords:** high-risk human papillomavirus, female genital schistosomiasis, neglected tropical diseases, sub-Saharan Africa, sexual and reproductive health

## Abstract

**Background:**

Female genital schistosomiasis (FGS), a gynecological disease caused by *Schistosoma haematobium* egg deposition in the female genital tract, is prevalent in sub-Saharan Africa, the region with the highest cervical cancer burden globally. Persistent high-risk human papillomavirus (HR-HPV) infection is necessary for cervical cancer development. We determined the association between FGS and HR-HPV genotypes in Zambian women.

**Methods:**

Sexually active women aged 15–50 years, not menstruating or pregnant, were recruited at home and provided 2 cervicovaginal self-swabs, urine sample, and HIV and *Trichomonas vaginalis* self-tests, and completed a questionnaire. In clinic, midwives collected cervicovaginal swabs and cervical images with point-of-care colposcopy (EVA System, MobileODT). Swabs were analyzed for 14 HR-HPV types (GeneXpert) and *Schistosoma* DNA (ITS-2 real-time polymerase chain reaction [qPCR]); urine was analyzed for *Schistosoma* ova by microscopy and circulating anodic antigen. Visual FGS was defined as colposcopic identification of specific genital lesions, and molecular FGS as *Schistosoma-*positive cervicovaginal qPCR.

**Results:**

Among 2532 women (median age, 28 [interquartile range, 22–36] years) recruited at home, 1694 (67%) completed clinic follow-up. Prevalence of visual FGS, molecular FGS, and HR-HPV was 35.2% (595/1691), 6.5% (165/2532), and 28.7% (690/2401), respectively. Molecular FGS was weakly associated with all HR-HPV (adjusted odds ratio [aOR], 1.3 [95% confidence interval {CI}, .9–1.9]). Women with molecular FGS were more likely to test positive for HR-HPV types 16/18/45 (aOR, 1.7 [95% CI, 1.0–2.8]). No association was observed between visual FGS and HR-HPV infection (95% CI, .7–1.1).

**Conclusions:**

This is the first study to jointly screen for FGS and HR-HPV in Zambia, reporting an association between oncogenic HR-HPV types and molecular FGS.

## Teaser

Among 2532 women of reproductive age in Zambia, female genital schistosomiasis was associated with the most oncogenic high-risk human papillomavirus types. These findings support integrated screening approaches in schistosomiasis-endemic settings.

Female genital schistosomiasis (FGS) is a neglected gynecological disease estimated to affect 30–56 million women globally, mostly in sub-Saharan Africa [1, 2]. It is caused by *Schistosoma haematobium* egg deposition in the female genital tract, which induces an inflammatory response, the formation of granulomas, and epithelial damage presenting as characteristic cervicovaginal mucosal lesions [1, 2]. Untreated FGS is associated with sexual dysfunction and reproductive tract morbidity, including infertility, dyspareunia, and genital symptoms mimicking sexually transmitted infections (STIs) [2]. Despite its high burden, FGS remains underdiagnosed and overlooked in schistosomiasis and in sexual and reproductive health (SRH) programs [1, 2].

There are no guidelines for FGS screening, diagnosis, or treatment [2–4]. Conventional diagnosis involves colposcopy to collect cervicovaginal images, then analyzed by gynecologists and classified as suggestive of “visual FGS” if characteristic cervicovaginal mucosal findings (homogeneous sandy patches, grainy sandy patches, rubbery papules, or abnormal blood vessels) are observed [2–5]. This requires expensive equipment, advanced clinical infrastructure, and specialized training, often not available in *S haematobium–*endemic settings [2, 6]. In addition, concordance between expert reviewers is poor (Cohen κ = 0.16), highlighting the low specificity of visual FGS diagnosis [6]. Molecular testing using polymerase chain reaction (PCR) to detect *S haematobium* DNA in genital swabs (“molecular FGS”) offers a potentially scalable and specific diagnostic method [3, 7]. Genital samples can be self-collected at home, potentially increasing screening coverage [3, 7].

Zambia has the third-highest incidence and mortality rates of cervical cancer globally, with 65.5 new cases and 43.4 cancer deaths per 100 000 women annually [8–11]. Persistent infection with 1 of the 12 high-risk human papillomavirus (HR-HPV) types (16, 18, 31, 33, 35, 39, 45, 51, 52, 56, 58, and 59) is a necessary cause of cervical cancer [12]. HPV-16 and -18 account for 70% of all cervical cancer cases [12, 13]. HR-HPV is primarily sexually transmitted, and risk factors include early sexual debut, multiple lifetime sexual partners, high parity, hormonal contraceptive use, and STI coinfection [14]. Women with human immunodeficiency virus (HIV-1) have 2-fold increased risk of acquiring HR-HPV [15], and 6-fold increased risk of cervical cancer, compared to women without HIV [16, 17]. The chronic genital inflammation caused by *S haematobium* eggs may facilitate HR-HPV acquisition, persistence, and progression to cervical precancer, yet this requires further investigation [2, 18].

The World Health Organization (WHO) announced a global call to eliminate cervical cancer by 2030, setting targets to vaccinate 90% of girls against HPV by age 15, screen 70% of eligible women with high-performance tests, and treat 90% of women with cervical cancer [19]. Zambia launched its national HPV vaccination program in 2019 and is transitioning from visual inspection with acetic acid to HPV DNA–based screening, due to its higher sensitivity and specificity to detect cervical precancer [20]. HPV DNA testing can be performed on provider- or self-collected samples, with comparable sensitivity and specificity between the 2 methods [20–22]. Molecular testing for FGS could be integrated into the cervical cancer screening programs and delivered at home for multipathogen community-based screening [7, 8]. Understanding the epidemiological overlap between FGS and HR-HPV is essential to inform integrated screening strategies [7].

Previous studies exploring the association of FGS and HPV in sub-Saharan Africa showed mixed results [2, 18]. A study in Zimbabwe among 236 women (aged 15–49 years) showed an association between composite FGS diagnosis by visual and molecular methods and HR-HPV infection (age-adjusted odds ratio [OR], 1.9 [95% confidence interval {CI}, 1.1–3.6], *P* = .032) [23]. Another South African study (N = 933) found strong association between visual FGS and any HR-HPV infections (detected by GP5+/6+ HPV PCR test) (adjusted OR [aOR], 1.71 [95% CI, 1.14–2.56], *P* = .010) [24]. In contrast, a study in Madagascar (N = 302) found no association between visual FGS and infection with any HPV (high- and low-risk genotypes) (OR, 1.0 [95% CI, .82–1.2]) [25]. A study in Tanzania found no differences in HR-HPV rates between women with and without *Schistosoma* infection, detected by circulating anodic antigen (CAA), an indirect measure that does not indicate genital involvement [26].

To date, no study has evaluated the association between FGS diagnosed using both visual and molecular diagnostic methods and individual HR-HPV genotypes. This study aims to determine the risk factors for HR-HPV and the association of HR-HPV prevalence and FGS diagnosed by visual and molecular diagnostic methods, in an ongoing longitudinal cohort in Zambia (Zipime-Weka-Schista study).

## MATERIALS AND METHODS

### Study Design and Participants

The Zipime-Weka-Schista (Do-self-testing sister!) study is a 4-year longitudinal cohort study evaluating the performance of multipathogen self-sampling for genital infections among women in Zambia [27]. This analysis uses baseline data collected between January 2022 and April 2023, across 3 sites (Livingstone, Sikoshwe, and Chanyanya). Women aged 15–50 years, who were nonpregnant, nonmenstruating, sexually active, and resident for at least a year in the study sites, were eligible. Participants were randomly selected through community-based random sampling and enrolled at home by Schista community workers (SCWs) [27].

### Sample Collection

During home visits, participants provided written informed consent, completed a questionnaire, and self-collected 2 cervicovaginal swabs and a urine specimen [27]. One swab was stored in PrimeStore molecular transport media (MTM; donated by Longhorn Vaccines and Diagnostics LLC, Bethesda, MD, USA) for *Schistosoma* DNA detection (Supplementary Text 1) [27]. The second swab was stored in PrimeStore MTM (80% of baseline samples) or in ThinPrep Cytopreserve solution (Supplementary Text 1). Urine was tested on the same day on-site for *S haematobium* egg-patent infection by urinary microscopy (Supplementary Text 2). A 2-mL urine sample was shipped to Leiden University Medical Centre, The Netherlands, for *Schistosoma* CAA detection using the up-converting reporter particle lateral flow CAA test (UCAA*hT*417 format; CAA levels >0.6 pg/mL were considered positive) (Supplementary Text 3) [28]. Participants were offered HIV (OraQuick HIV Self-Test) and *Trichomonas vaginalis* self-tests (OSOM Trichomonas Rapid Test). Results were read by the SCW and immediately shared with the participant. HIV-positive self-tests were confirmed on-site using a point-of-care diagnostic test (Determine) [27]. Participants with positive HIV or *T vaginalis* results were referred for treatment and care.

Following the home visit, participants were invited for the clinic follow-up where a midwife collected 2 cervicovaginal swabs, a cervicovaginal lavage (CVL), and cervicovaginal images with a point-of-care colposcope (EVA MobileODT, Tel Aviv, Israel). One cervicovaginal swab was stored in PrimeStore MTM and the second in Cytopreserve. The CVL specimen was stored at −20°C for future analysis (Supplementary Text 1).

#### Visual FGS

Colposcopic cervicovaginal images were independently evaluated remotely by 2 gynecologists (B. R. and D. K.). In case of disagreement, a third reviewer (I. H.) independently analyzed the images. Participants were classified as visual FGS positive if at least 2 reviewers independently identified at least 1 of the 4 typical FGS lesion types, and negative if no lesions were observed [5].

#### Molecular FGS

Cervicovaginal swabs stored in PrimeStore MTM were processed at Zambart central laboratory (Lusaka, Zambia) for *Schistosoma* DNA detection using internal transcribed spacer 2 (ITS-2) by real-time PCR (ITS qPCR) (Supplementary Text 4). Total nucleic acid extraction (PrimeXtract Longhorn Vaccines and Diagnostics) was performed as per manufacturer's instructions, using a simplified silica-based spin column extraction process. qPCR was performed as previously reported and results were reported as cycle threshold (Ct) values and considered positive if any Ct value was observed and negative if no Ct value was observed [3, 29]. Women were classified as molecular FGS positive if either self- or provider-collected cervicovaginal swab tested positive. Women with evidence of schistosomiasis, molecular FGS, and/or visual FGS received 40 mg/kg praziquantel single dose [30].

### HR-HPV Detection

Self-collected cervicovaginal swabs stored in either PrimeStore MTM or in ThinPrep Cytopreserve were tested for HR-HPV detection using a multiplex real-time PCR assay (GeneXpert HPV, Cepheid, USA), which simultaneously detects 14 HR-HPV types (HPV types 16, 18, 45, 31, 33, 35, 52, 58, 51, 59, 39, 56, 66, and 68), grouped into 3 channels: HPV-16; HPV-18 or -45 (or both); and any other HR-HPV types. DNA extraction for samples stored in PrimeStore MTM was performed using a modified PrimeXtract spin column protocol (Supplementary Text 5). For GeneXpert HPV analysis, 50 µL of the extracted DNA was diluted in 1 mL of normal saline and loaded into the GeneXpert cartridge, following the standard assay protocol. A sample adequacy control and a probe check control was also provided in the cartridge (Supplementary Text 5). If a test result on the self-collected swab was invalid, HR-HPV testing was done using the healthcare provider-collected swab (Supplementary Text 6).

### HR-HPV Definitions

Participants were classified as HR-HPV positive if positive for at least 1 of 14 HR-HPV types; HPV-16/18/45 positive if positive for HPV type 16, and/or HPV type 18 and/or 45 channels; and HPV-16 positive if only HPV type 16 channel was positive (Supplementary Figure 1). Samples were classified as invalid if both the home-based self-collected swab and clinic-based swab were invalid (Supplementary Text 6). HR-HPV–positive women were referred to a gynecologist for colposcopy and a 2-quadrant cervical biopsy according to age and HIV status [27]. All women with cervical precancer were referred for management as per national guidelines [20].

### Statistical Analyses

Data were entered on electronic devices using Open Data Kit and analyzed using Stata version 17.0 software (StataCorp, USA). Primary outcomes were detection of any HR-HPV, HPV-16/18/45, and HPV-16 only. Descriptive statistics summarized participants’ characteristics, stratified by outcomes. Univariable logistic regression analyses were used to estimate crude ORs (cORs) and 95% CIs for the associations between HR-HPV and potential risk factors. Potential risk factors were informed by the published literature. Multivariable logistic regression models were used to estimate aORs for the associations between HR-HPV and factors associated with HR-HPV in univariable analysis. Covariates with missing data were excluded. Multicollinearity was assessed between variables in the final models.

### Ethical Considerations

The study was approved by the University of Zambia Biomedical Research Ethics Committee (UNZABREC) (reference: 1858-2021), the Zambian National Health Research Authority (Reference: 00012/24/09/2021), and the London School of Hygiene and Tropical Medicine (LSHTM) (reference: 25258).

## RESULTS

**Figure 1.**
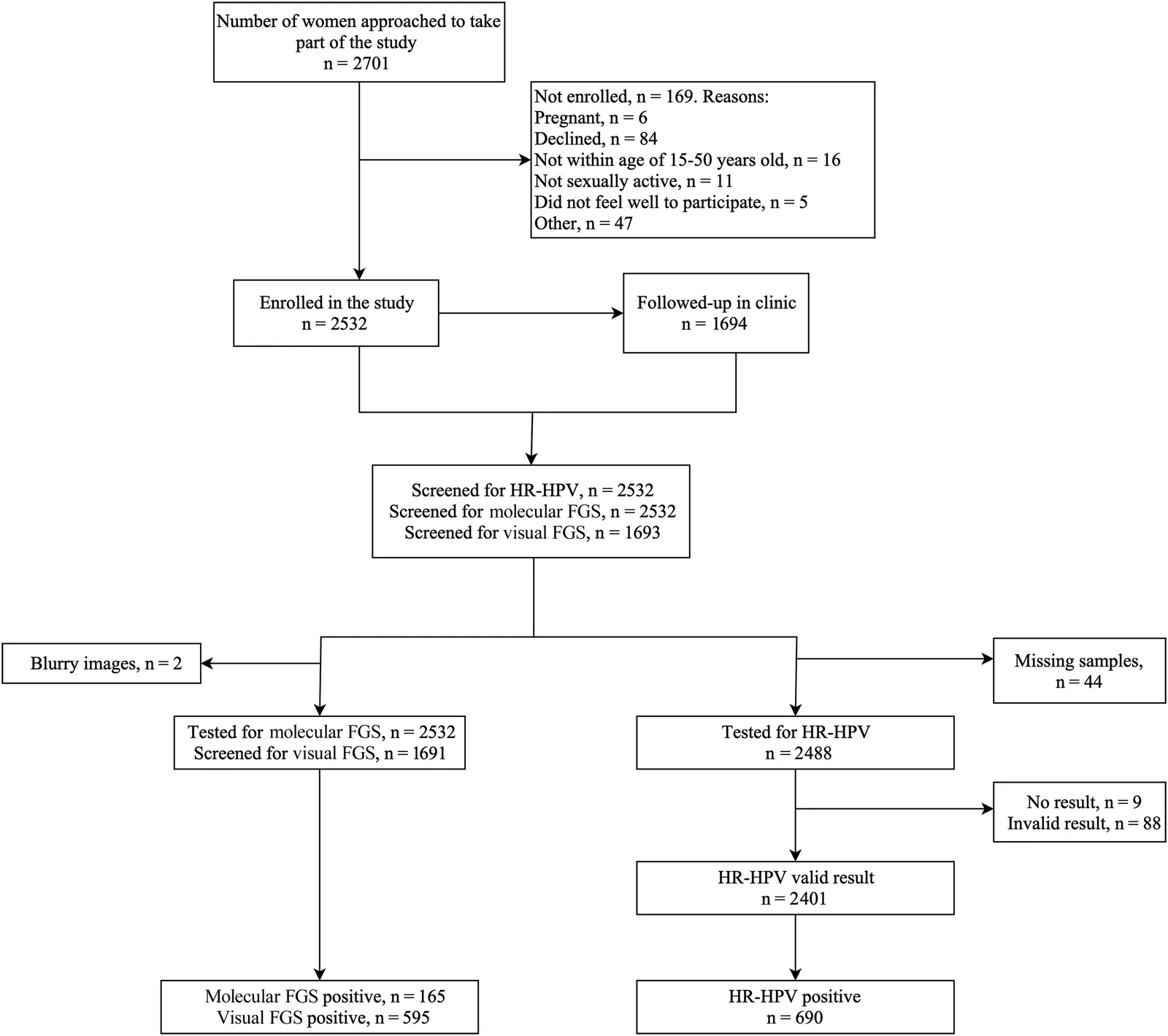
Flow diagram showing participant recruitment at baseline for the Zipime-Weka-Schista study and screening of molecular female genital schistosomiasis (FGS), visual FGS, and high-risk human papillomavirus (HR-HPV). 2,701 women were approached to take part of the study at home, 2,532 were enrolled in the study and 1,694 completed clinic follow-up. All enrolled women were screened for molecular FGS and HR-HPV; 1,693 were screened fro visual FGS. Among the study population, 165 women tested positive for molecular FGS, 595 for visual FGS and 690 for HR-HPV.

At baseline, 2532 women (median age, 28 years [interquartile range {IQR}, 22–36 years]) were enrolled at home, and 67% (1694/2532) completed clinic follow-up (Figure 1). All urine samples were tested for *S haematobium* ova by microscopy, and 99.4% (2517/2532) were tested for CAA. Visual FGS was available for 99.8% (1691/1694) of participants. HR-HPV DNA testing was performed on 98.3% (2488/2532) of participants, and 96.5% (2401/2488) gave valid HR-HPV results. Molecular FGS diagnosis was available for all participants (Figure 1). Data for both HR-HPV and molecular FGS status were available for 94.8% (2401/2532) of participants, and for HR-HPV and visual FGS in 96.7% (1639/1694) of clinic attendees.

### Participant Characteristics

Of 2532 women, 165 (6.5%) tested positive for molecular FGS (median Ct value, 35.1 [IQR, 7.4–31.6]) (Table 1). Prevalence of visual FGS was 35.2% (595/1691). Among women with molecular FGS, 39.0% (46/165) were visual FGS positive. Of the 595 women with visual FGS, 46 (7.7%) were also diagnosed with molecular FGS. Prevalence of egg-patent *S haematobium* infection was 5.2% (132/2532) (mean intensity: 4.8 eggs/10 mL of urine; standard deviation [SD], 5.5) and *Schistosoma* CAA was 15.4% (388/2517) (median CAA concentration, 4.6 pg/mL [IQR, 1.1–31.0 pg/mL]). HIV prevalence was 17.5% (443/2532), and 91.6% (406/443) self-reported antiretroviral therapy (ART) use. *Trichomonas vaginalis* prevalence was 10.3% (246/2389).

**Table 1.** Prevalence of Any High-Risk Human Papillomavirus Stratified by Sociodemographic Characteristics and Coinfections, With Univariable Logistic Regression Estimates Across the Study Population (N = 2401)

Characteristic	All	HR-HPV Negative	HR-HPV Positive	Crude OR (95% CI)	*P* Value
Total	2532 (100)	1711 (71.3)	690 (28.7)	…	
Sociodemographic characteristics					
Age, y					
15–19	330 (13.0)	199 (64.0)	112 (36.0)	2.2 (1.3–3.5)	<.001
20–25	674 (26.6)	412 (64.9)	223 (35.1)	2.1 (1.3–3.3)	
26–30	553 (21.8)	380 (72.1)	147 (27.9)	1.5 (.9–2.4)	
31–35	341 (13.5)	245 (75.9)	78 (24.2)	1.2 (.8–2.0)	
36–40	293 (11.6)	223 (80.2)	55 (19.8)	1.0 (.6–1.6)	
41–45	200 (7.9)	144 (75.4)	47 (24.6)	1.3 (.7–2.1)	
46–50	141 (5.6)	108 (79.4)	28 (20.6)	Ref	
Marital status					
Single	330 (13.0)	559 (63.1)	327 (36.9)	2.0 (1.6–2.4)	<.001
Married	674 (26.6)	1023 (77.0)	306 (23.0)	Ref	
Divorced/separated	553 (21.8)	92 (71.9)	36 (28.1)	1.3 (.9–2.0)	
Widowed	341 (13.5)	36 (63.2)	21 (36.8)	2.0 (1.1–3.4)	
Education					
No schooling	49 (1.9)	36 (78.3)	10 (21.7)	Ref	.21
Some/completed primary school	909 (35.9)	620 (72.6)	234 (27.4)	1.4 (.7–2.8)	
Some/completed secondary school	1344 (53.1)	898 (70.2)	381 (29.8)	1.5 (.8–3.1)	
Some/completed trade school or degree	229 (9.1)	156 (70.6)	65 (29.4)	1.5 (.7–3.2)	
District of residence					
Shikoshwe	1176 (46.5)	818 (72.1)	316 (27.9)	Ref	.44
Livingstone	1196 (47.2)	781 (70.4)	328 (29.6)	1.1 (.9–1.3)	
Chanyanya	160 (6.3)	112 (70.9)	46 (29.1)	1.1 (.7–1.5)	
Sexual and reproductive health behaviors and outcomes					
Number of sexual partners in the last 6 mo					
0	131 (5.2)	88 (69.8)	38 (30.2)	Ref	.49
1	2144 (84.7)	1457 (71.7)	574 (28.3)	0.9 (.6–1.4)	
2	152 (6.0)	96 (67.1)	47 (32.9)	1.1 (.7–1.9)	
≥2	105 (4.2)	70 (69.3)	31 (30.7)	1.0 (.6–1.8)	
Age of sexual debut					
≤15 y	477 (18.9)	301 (68.1)	141 (31.9)	Ref	.03
16–20 y	1768 (70.0)	1205 (71.3)	485 (28.7)	0.9 (.7–1.1)	
>20 y	282 (11.2)	200 (75.8)	64 (24.2)	0.7 (.5–1.0)	
Time to conception of previous pregnancy					
0–3 mo	401 (19.2)	277 (74.1)	97 (25.9)	Ref	.4
4–6 mo	95 (4.5)	66 (69.5)	29 (30.5)	1.3 (.8–2.1)	
10–12 mo	1038 (49.6)	722 (73.7)	258 (26.3)	1.0 (.8–1.3)	
Don’t remember	31 (1.5)	24 (80.0)	6 (20.0)	0.7 (.3–1.8)	
Pregnancy was unplanned	529 (25.3)	354 (70.0)	152 (30.0)	1.2 (.9–1.7)	
Number of pregnancies					
Never pregnant	437 (17.3)	267 (64.3)	148 (35.7)	1.8 (1.4–2.5)	<.001
1	551 (21.8)	335 (64.6)	184 (35.5)	1.8 (1.4–2.5)	
2	431 (17.0)	289 (71.0)	118 (29.0)	1.4 (1.0–1.9)	
3	402 (15.9)	287 (74.4)	99 (25.7)	1.1 (.8–1.6)	
4	289 (11.4)	227 (82.3)	49 (17.8)	0.7 (.5–1.1)	
>4	422 (16.7)	306 (76.9)	92 (23.1)	Ref	
Type of contraception used					
None	10 (0.8)	7 (77.8)	2 (22.2)	Ref	.41
Barrier method (condom, diaphragm, or IUD)	139 (11.4)	101 (74.8)	34 (25.2)	1.2 (.2–5.9)	
Hormonal method (implant, injectable, or oral pill)	999 (82.1)	697 (71.7)	275 (28.3)	1.4 (.3–6.7)	
Sterilization method	1 (0.1)	1	…	Not enough data	
Combination (hormonal and barrier)	69 (5.7)	46 (68.7)	21 (31.3)	1.6 (.3–8.4)	
Number of stillbirths					
None	1978 (94.5)	1360 (72.6)	513 (27.4)	Ref	.006
1 or more	116 (5.5)	351 (66.5)	177 (33.5)	1.3 (1.1–1.6)	
Coinfections					
HIV status					
Negative	1970 (77.8)	1330 (71.4)	534 (28.7)	Ref	.74
Positive	443 (17.5)	304 (71.7)	120 (28.3)	1.0 (.8–1.2)	
Unknown	119 (4.7)	77 (68.1)	36 (31.9)	1.2 (.8–1.8)	
Self-reported ART use among HIV positive (n = 443)					
No	38 (8.6)	20 (54.1)	17 (45.7)	2.3 (1.2–2.4)	.02
Yes	405 (91.4)	284 (73.4)	103 (26.6)	Ref	
*Trichomonas vaginalis* status					
Negative	2143 (89.7)	1444 (71.5)	575 (28.5)	Ref	.15
Positive	246 (10.3)	161 (67.1)	79 (32.9)	1.2 (.9–1.6)	
Molecular FGS^a^					
Negative	2366 (93.5)	1610 (71.8)	633 (28.2)	Ref	.04
Positive	165 (6.5)	101 (63.9)	57 (36.1)	1.5 (1.0–2.0)	
Median Ct (IQR)	35.1 (7.4–31.6)	35.7 (31.3–39.0)	35.1 (31.8–38.7)	…	
Visual FGS by colposcopy^b^					
Negative	1096 (64.8)	729 (68.8)	331 (31.2)	Ref	.17
Positive	595 (35.2)	417 (72.0)	162 (28.0)	0.9 (.7–1.1)	
*Schistosoma* infection by CAA					
Negative	2129 (84.6)	1437 (71.2)	580 (28.8)	Ref	0.99
Positive	388 (15.4)	263 (71.3)	106 (28.7)	1.0 (.8–1.3)	
Median pg/mL^c^ (IQR)	4.6 (1.1–30.6)	3.4 (1.0–26.7)	10.2 (1.2–50.3)	…	
*Schistosoma haematobium* by microscopy					
Negative	2400 (94.8)	1635 (71.9)	639 (28.1)	Ref	.004
Positive	132 (5.2)	76 (59.8)	51 (40.2)	1.7 (1.2–2.5)	
Mean egg count (SD) per 10 mL^c^	4.8 (5.5)	4.9 (4.8)	4.8 (6.7)	…	

Data are presented as No. (%) unless otherwise indicated. Percentages (%) in the first column are based on the total number of observations (N = 2532). Percentages (%) in the second and third columns are calculated within each row total.

Abbreviations: ART, antiretroviral therapy; CAA, circulating anodic antigen; CI, confidence interval; Ct, cycle threshold; FGS, female genital schistosomiasis; HIV, human immunodeficiency virus; HR-HPV, high-risk human papillomavirus; IQR, interquartile range; IUD, intrauterine device; OR, odds ratio; SD, standard deviation.

^a^Molecular FGS refers to polymerase chain reaction (PCR) diagnosis of either home-based self-collected cervicovaginal swabs or clinic-based provider-collected cervicovaginal swabs.

^b^Visual FGS defined as a positive if confirmed by both reviewers 1 and 2, or in case of disagreement, by reviewer 3. The associations between the single visual FGS and HR-HPV infection are presented in Supplementary Table 2.

^c^Mean egg count, CAA concentrations, and PCR Ct values are restricted to positive tests.

### Distribution of Infection by Age

**Figure 2.**
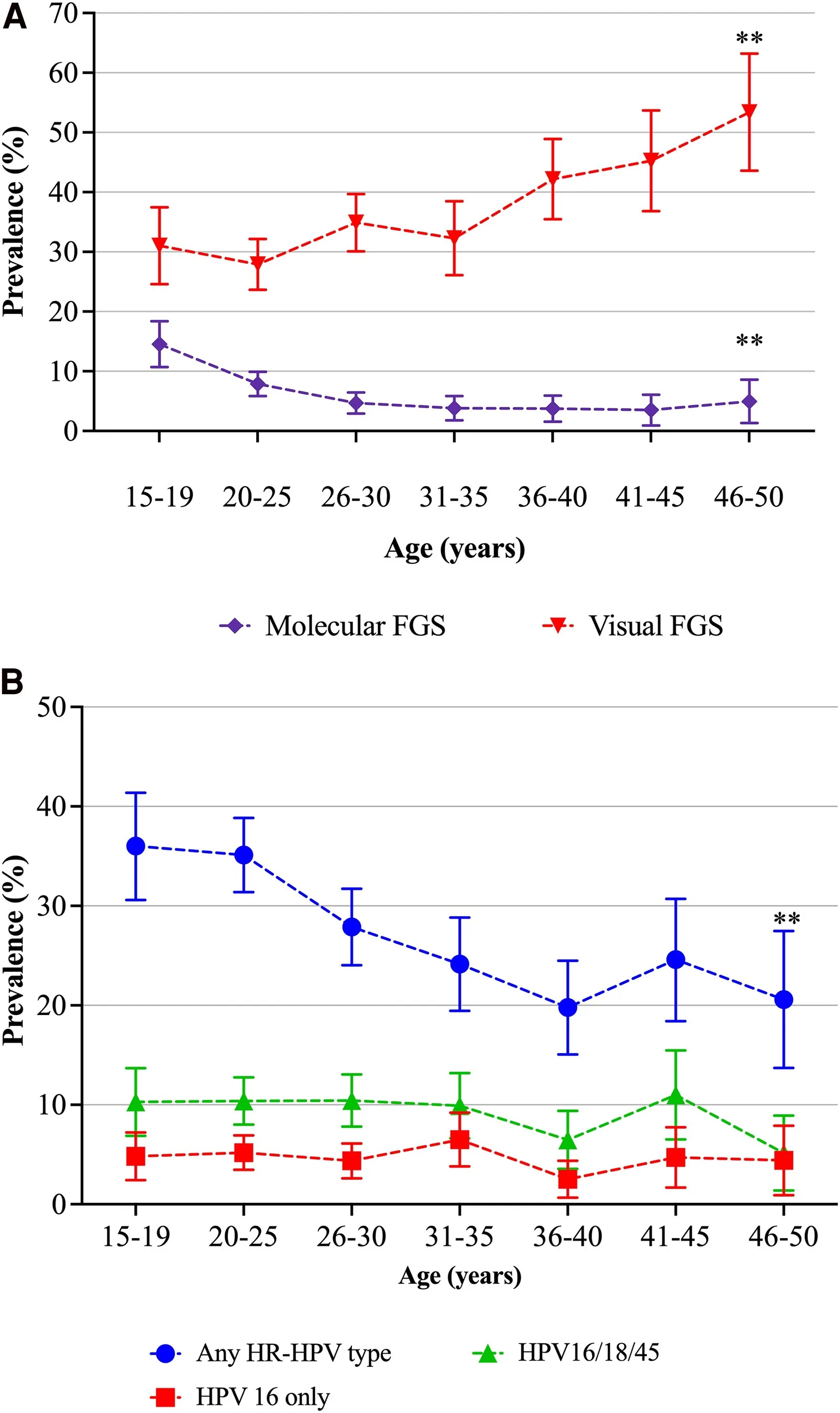
Two-panel plot showing the distribution of positive diagnostic test results by age categories. Panel A shows the distribution of molecular female genital schistosomiasis (FGS) and visual FGS by age groups. Panel B shows the prevalence of any high-risk human papillomavirus (HR-HPV) infection, HPV-16/18/45, and HPV-16 only by age categories. The corresponding data are presented in Table 1 and Supplementary Table 1. **Stands for *P*-value < .01.

Prevalence of molecular FGS was highest among adolescents aged 15–19 years (14.6%) and declined with age (χ^2^ < 0.001) (Figure 2, Supplementary Table 1). Visual FGS prevalence increased with age, peaking at 46–50 years (53.4%) (χ^2^ < 0.001; Figure 2, Supplementary Table 1). HR-HPV prevalence declined significantly with age (*P* < .001) (Figure 2, Table 1).

### Risk Factor Analysis for Prevalence of Any HR-HPV

**Figure 3.**
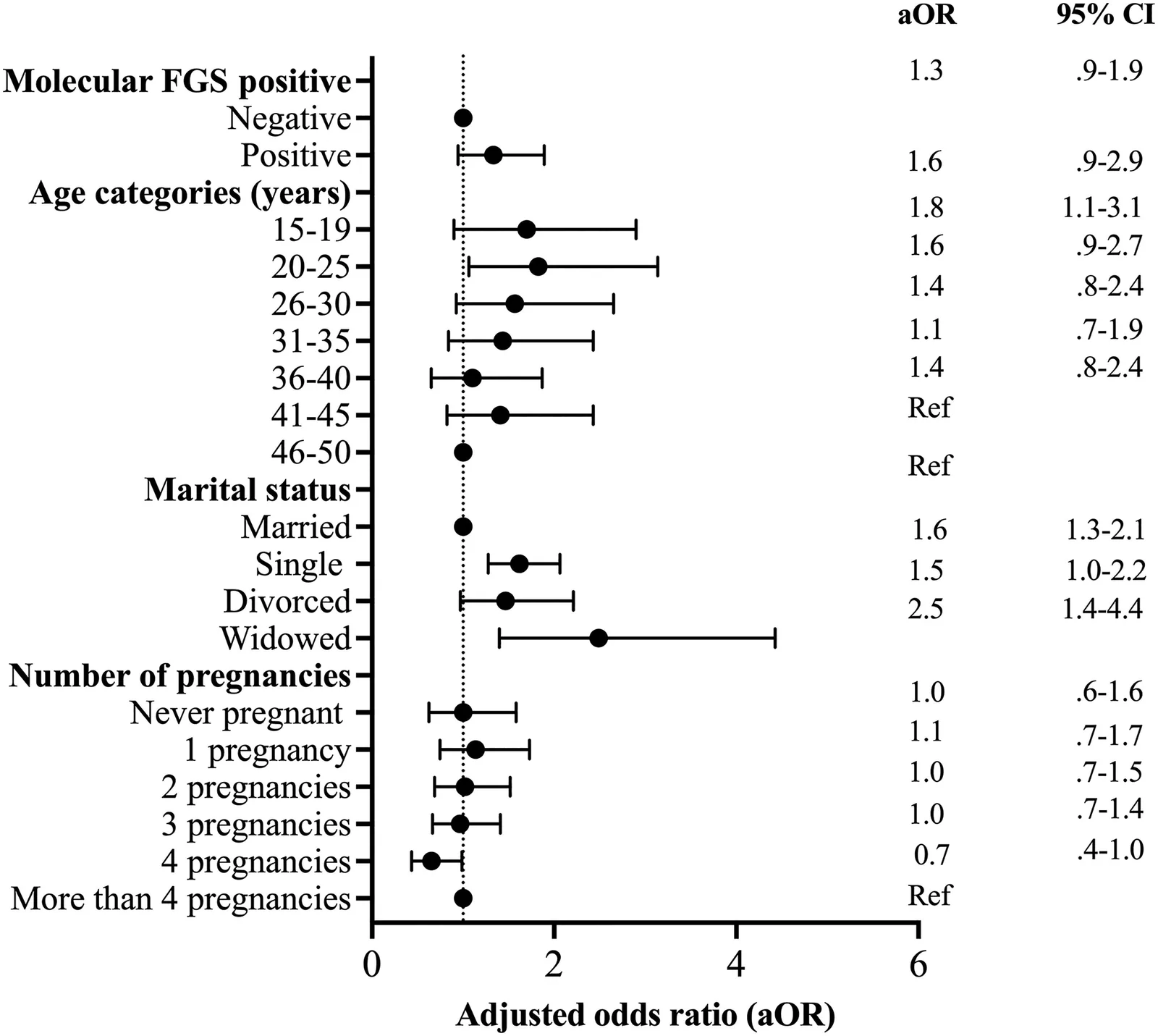
Forest plot showing adjusted odds ratios (aOR) and 95% confidence intervals for risk factors associated with prevalence of any high-risk human papillomavirus across the study population (N = 2,401). The regression model was adjusted for molecular female genital schistosomiasis status, age, marital status, and number of pregnancies. Abbreviations: aOR, adjusted odds ratio; CI, confidence interval; FGS, female genital schistosomiasis.

Prevalence of any HR-HPV genotypes was 28.7% (690/2401) (Table 1). Any HR-HPV prevalence was higher among women with molecular FGS compared to those without (36.1% vs 28.2%; cOR, 1.5 [95% CI, 1.0–2.0]) (Table 1). No statistically significant difference was observed by visual FGS status (visual FGS positive vs negative, 28.0% vs 31.2%; cOR, 0.9 [95% CI, .7–1.1]) (Table 1, Supplementary Table 2). Women with urinary *S haematobium* by microscopy had a higher prevalence of any HR-HPV compared to those without (40.2% vs 28.1; cOR, 1.7 [95% CI, 1.2–2.5]). No significant differences in HR-HPV prevalence were observed by HIV status (cOR, 1.0 [95% CI, .8–1.2]) or *T vaginalis* status (cOR, 1.2 [95% CI, .9–1.6]) (Table 1). After adjusting for age, marital status, and number of pregnancies, molecular FGS was weakly associated with any HR-HPV prevalence (aOR, 1.3 [95% CI, .9–1.9]) (Figure 3). Due to multicollinearity between molecular FGS and urinary *S haematobium* infection (*r* = 0.56), a separate model evaluated the association between urinary *S haematobium* infection by microscopy and infection with any HR-HPV (aOR, 1.7 [95% CI, 1.1–2.4]) (Supplementary Table 3).

### Risk Factor Analysis for Infection With High-Risk Types

#### HPV-16/18/45

**Figure 4.**
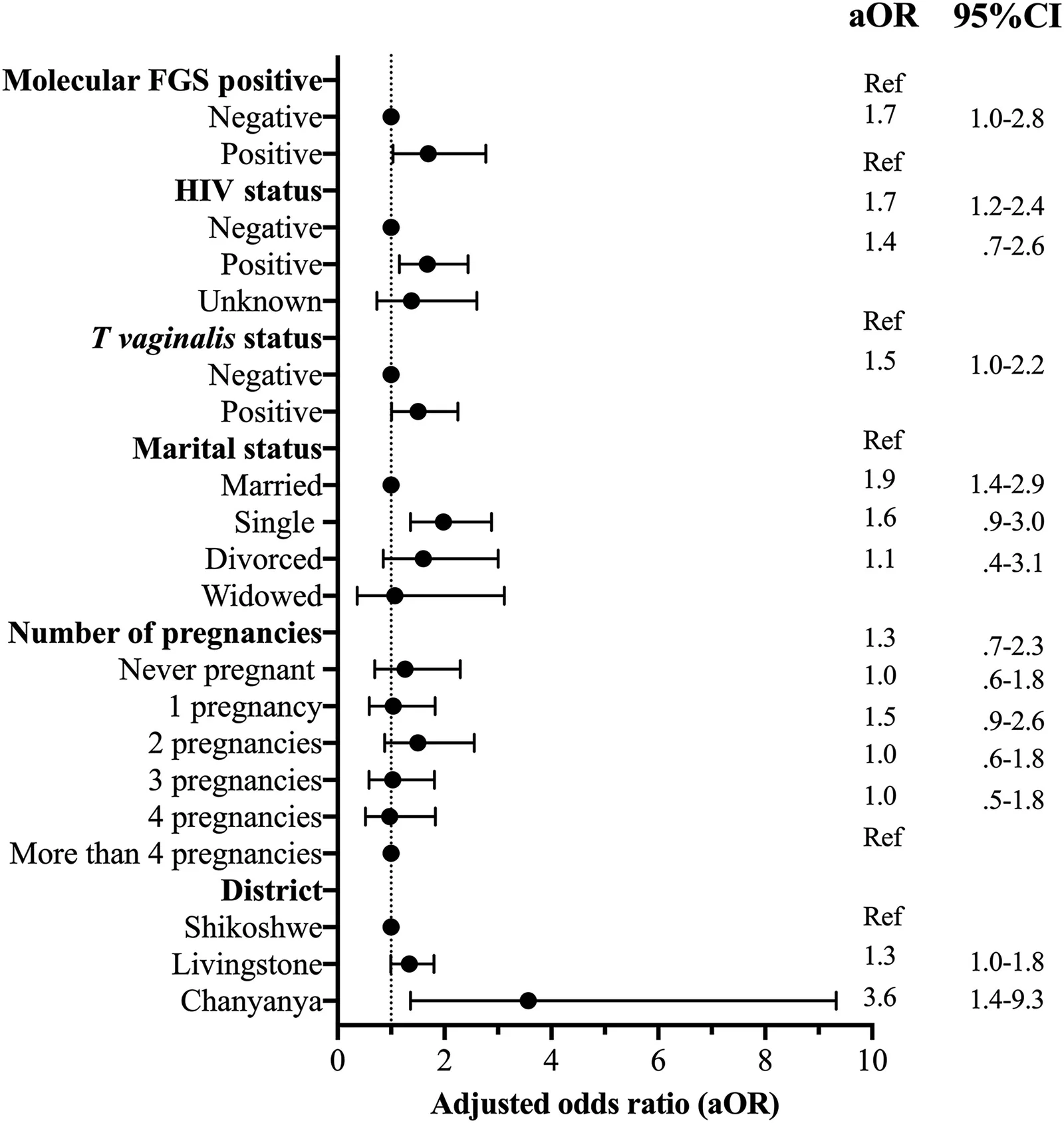
Forest plot showing adjusted odds ratios (aOR) and 95% confidence intervel for risk factors associated with the prevalence of human papillomavirus type 16/18/45 across the study population (N = 2,401). The corresponding data are presented in Supplementary Table 4. The model was adjusted for molecular female genital schistosomiasis status, HIV status, *Trichomonas vaginalis* status, district of residence, marital status, and number of pregnancies. Abbreviations: aOR, adjusted odds ratio; CI, confidence interval; FGS, female genital schistosomiasis; HIV, human immunodeficiency virus.

Prevalence of infection with HPV-16/18/45 was 9.6% (231/2401) (Supplementary Table 4). In univariable analysis, higher prevalence was observed among HIV-positive versus HIV-negative women (12.5% [53/424] vs 8.9% [166/1864]; cOR, 1.5 [95% CI, 1.1–2.0]) and among women with *T vaginalis* (14.6% [35/240] vs 8.8% [177/2019]; cOR, 1.8 [95% CI, 1.2–2.6]) (Supplementary Table 4). HPV-16/18/45 prevalence was higher among women with molecular FGS compared to those without (15.8% [25/158] vs 9.2% [206/2243]; cOR, 1.9 [95% CI, 1.2–2.9]). No association was observed with visual FGS (cOR, 0.8 [95% CI, .6–1.2]) (Supplementary Tables 4 and 5). In adjusted analysis, molecular FGS (aOR, 1.7 [95% CI, 1.0–2.8]), HIV status (aOR, 1.7 [95% CI, 1.2–2.4]), and *T vaginalis* status (aOR, 1.5 [95% CI, 1.0–2.2]) were associated with increased odds of HPV-16/18/45 infection (Figure 4).

#### HPV-16 Only

**Figure 5.**
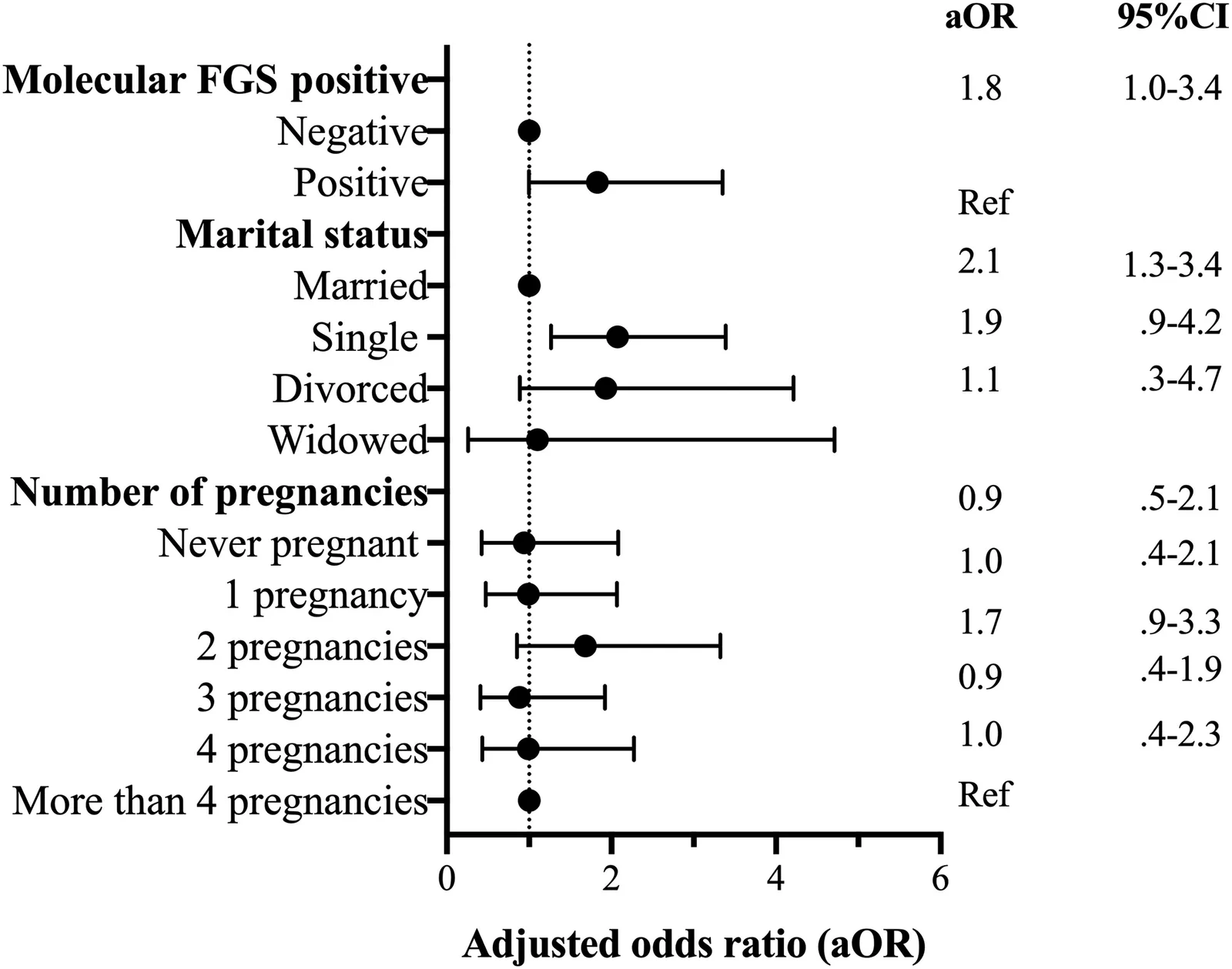
Forest plot showing adjusted odds ratios (aOR) and 95% confidence intervals for factors associated with the prevalence of human papillomavirus type 16 across the study population (N = 2,401). The corresponding data are presented in Supplementary Table 6. The logistic regression model was adjusted for molecular female genital schistosomiasis status, marital status, and number of pregnancies. Abbreviations: aOR, adjusted odds ratio; CI, confidence interval; FGS, female genital schistosomiasis.

HPV-16 prevalence was 4.8% (114/2401) (Supplementary Table 6). HPV-16 prevalence was higher among single versus married women (6.6% [58/886] vs 3.5% [46/1329]; cOR, 2.0 [95% CI, 1.3–2.9]) (Supplementary Table 6). Women with molecular FGS had higher HPV-16 prevalence compared to those without (8.2% [13/158] vs 4.5% [101/2243]; cOR, 1.9 [95% CI, 1.0–3.5]) (Supplementary Table 6). No significant association was observed with visual FGS status (cOR, 0.8 [95% CI, .5–1.2]), urinary *S haematobium* status by microscopy (cOR, 1.4 [95% CI, .7–2.9]), *Schistosoma* status by CAA (cOR, 1.2 [95% CI, .8–2.0]), HIV status (cOR, 1.4 [95% CI, .9–2.2]), or *T vaginalis* status (cOR, 1.4 [95% CI, .8–2.5]) (Supplementary Tables 6 and 7). After adjusting for marital status and parity, women with molecular FGS had 1.8 higher odds of being HPV-16 positive compared to those without (aOR, 1.8 [95% CI, 1.0–3.4]) (Figure 5).

## DISCUSSION

To our knowledge, the Zipime-Weka-Schista study is the first to investigate the association between HR-HPV and FGS, diagnosed using both visual and molecular methods, in a large cohort of women with and without HIV in Zambia [27]. We found a significant association between molecular FGS and HPV-16/18/45, the types most frequently associated with cervical precancer and cancer. No significant association was found between visual FGS and HR-HPV infection. These findings highlight the need to recognize FGS as an SRH condition and integrate FGS screening into cervical cancer control programs to reduce the dual burden of diseases in endemic settings [7].

Women with molecular FGS were almost 1.7 times more likely to test positive for HPV-16/18/45, the most oncogenic genotypes, which contribute to the majority of cervical precancer and cancer globally [12, 13]. To our knowledge, this is the first study to compare molecular FGS with HR-HPV infection from cervicovaginal self-collected swabs. FGS may influence the natural history of cervical HPV infection. HPV infects undifferentiated, proliferating basal cells of the cervicovaginal epithelium through microabrasion, often resulting from coexistence with STIs or co-factors that compromise the epithelial barrier [31]. The epithelial damage of the anogenital mucosa caused by *S haematobium* egg deposition in women with FGS may facilitate HPV viral entry [18]. Further, effective immune regulation is critical for HR-HPV clearance, and chronic genital inflammation associated with *S haematobium* egg deposition may impair local immune responses, promoting immune evasion and HR-HPV persistence [18, 31]. Although this cross-sectional analysis does not allow to distinguish between incident and persistent HR-HPV infections, the observed association between molecular FGS and HPV-16/18/45 support the hypothesis that FGS may contribute to HPV persistence. This aligns with a study in Tanzania reporting higher odds of HR-HPV persistence among women with *Schistosoma* infection diagnosed by CAA [26]. Although genital involvement was not assessed, it suggests that the schistosomiasis-related inflammation may promote HR-HPV persistence [26]. Longitudinal data from the ongoing Zipime-Weka-Schista study will further evaluate whether FGS affects HR-HPV persistence or clearance [27].

Women with egg-patent urinary *S haematobium* infection were more likely to have prevalent HR-HPV infection, compared to those without. In contrast, no significant association was observed with schistosomiasis detected by CAA. This may reflect the differences between diagnostic method [30]. CAA is a highly sensitive for active *Schistosoma* infection, detecting antigens released by live adult worms in the host's bloodstream [28]. However, it does not provide information on egg deposition or differentiate between species [28]. The associations observed highlight the complexity of active infection in the pathogenesis of HR-HPV in the presence of *S haematobium.*

Visual FGS was not associated with HR-HPV prevalence. Previous studies reported mixed results, likely due to differences in outcome definitions [24, 25]. Although colposcopy is widely used to assess FGS-related morbidity, its scalability in endemic settings is limited by high cost, infrastructure requirements, and need for trained personnel [2, 6]. Importantly, the mucosal changes in visual FGS*–*positive women can be similar to changes from other SRH, including cervical lesions caused by HPV, resulting in limited diagnostic specificity [6, 7]. In our study, agreement between 2 expert reviewers in detecting visual FGS was low, consistent with previous research in Zambia, highlighting the need for standardized, scalable diagnostic methods [6]. Emerging computer vision technologies, powered by artificial intelligence, are being explored for visual FGS diagnosis and may be integrated with tools being validated for automated detection of cervical precancer and cancer [32–35].

HR-HPV prevalence was highest among persons aged 15–25 years, consistent with previous studies [36–39]. Prevalence of molecular FGS decreased with increasing age and peaked among adolescents aged 15–19 years, whereas more older women (aged 41–50 years) were positive for visual FGS. These findings are consistent with previous studies [3, 4, 29, 40]. This age distribution supports opportunities to integrate FGS into cervical cancer control programs [7]. As Zambia transitions from visual inspection with acetic acid to HPV DNA–based cervical screening programs, self-collection platform and laboratory infrastructures for cervical cancer screening could include molecular FGS testing, improving earlier detection [7, 20]. Younger women are more susceptible to HR-HPV infection, and have higher intensity of *S haematobium* infection and higher rates of *Schistosoma* DNA retrieval from the genital tract [3, 29, 36–40]. In contrast, older women typically show higher prevalence of visually diagnosed lesions [3, 29, 40]. This age-specific pattern suggests that molecular FGS screening could be integrated into HPV vaccination and education programs targeting younger girls (9–14 years old), while community-based integrated HPV and molecular FGS screening can be introduced for women eligible for cervical cancer screening (aged ≥25 years for women with HIV or ≥30 years for women without HIV) [19, 20]. For older women (aged >35 years), FGS screening can be integrated into existing cervical cancer clinic-based screening and/or treatment programs [19, 20].

Prevalence of any HR-HPV did not vary by HIV status, differing from previous studies [41–43]. This may reflect the high proportion of participants on ART and younger age in our cohort. ART reduces HPV persistence and precancer and cancer incidence, especially when initiated early following HIV acquisition and before significant immunosuppression [17]. Yet, these benefits have primarily been demonstrated in cohorts where women achieve sustained viral suppression through long-term ART adherence and regular cervical cancer screening. In our community-based study, ART status was self-reported, and data on CD4^+^ T-cell count and HIV plasma viral load were unavailable, limiting our ability to assess immune control and its associations with HPV infection.

With >2500 women, this is the largest study to date to evaluate the association between HR-HPV prevalence and FGS, diagnosed using both molecular and visual methods. The integrated study design, combining home and clinic-based sampling with multiple diagnostic methods, allowed for comprehensive screening for FGS and SRH coinfections. There are some limitations worth noting. Histological examination of cervical tissues for *Schistosoma* eggs detection was not performed, limiting the ability to compare molecular and visual FGS diagnoses against a reference standard [2, 6, 44]. The reliability of visual FGS diagnosis was limited by low agreement between reviewers. Implementation of molecular FGS testing in routine settings is hindered by cost, technical requirements, and the need for trained personnel. Ct values may have been affected by the DNA extraction protocol, including the double-elution step, which can lower DNA concentration. Although this was applied consistently throughout the study, extraction variability may influence sensitivity and should be considered when interpreting absolute Ct values. This study accounted for HIV and *T vaginalis* status, but it did not address potential confounding by other STIs, which can affect cervical inflammation and epithelial integrity [45]. HIV status was self-reported for most participants, potentially introducing reporting bias. Data on CD4^+^ T-cell count, ART adherence and duration, and HIV plasma viral load were not collected, potentially introducing confounding. Further, the cross-sectional design does not distinguish between incident and persistent HR-HPV infections, limiting causal inferences. Longitudinal data from the ongoing Zipime-Weka-Schista study will address some of these limitations [27].

This study provides new evidence on the intersection of FGS, HR-HPV, and SRH among women of reproductive age, highlighting a significant association between molecular FGS and the most oncogenic HPV genotypes (HPV-16/18/45). In line with the Sustainable Development Goals and the WHO targets to eliminate cervical cancer and schistosomiasis, our findings support integrated strategies using highly specific, field-deployable, and cost-effective diagnostic methods.

## Supplementary Material

jiag153_Supplementary_Data

## Supplementary Data

Supplementary materials are available at *The Journal of Infectious Diseases* online (http://jid.oxfordjournals.org/). Supplementary materials consist of data provided by the author that are published to benefit the reader. The posted materials are not copyedited. The contents of all supplementary data are the sole responsibility of the authors. Questions or messages regarding errors should be addressed to the author.
